# Impact of innate immunity in a subset of children with autism spectrum disorders: a case control study

**DOI:** 10.1186/1742-2094-5-52

**Published:** 2008-11-21

**Authors:** Harumi Jyonouchi, Lee Geng, Agnes Cushing-Ruby, Huma Quraishi

**Affiliations:** 1 Division of Allergy/Immunology and Infectious Diseases, Department of Pediatrics, University of Medicine and Dentistry of New Jersey (UMDNJ)-New Jersey Medical School (NJMS), Newark, NJ, USA; 2 Department of Surgery, University of Medicine and Dentistry of New Jersey (UMDNJ)-New Jersey Medical School (NJMS), Newark, NJ, USA

## Abstract

**Background:**

Among patients with autism spectrum disorders (ASD) evaluated in our clinic, there appears to be a subset that can be clinically distinguished from other ASD children because of frequent infections (usually viral) accompanied by worsening behavioural symptoms and/or loss/decrease in acquired skills. This study assessed whether these clinical features of this ASD subset are associated with atopy, asthma, food allergy (FA), primary immunodeficiency (PID), or innate immune responses important in viral infections.

**Methods:**

This study included the ASD children described above (ASD test, N = 26) and the following controls: ASD controls (N = 107), non-ASD controls with FA (N = 24), non-ASD controls with chronic rhinosinusitis/recurrent otitis media (CRS/ROM; N = 38), and normal controls (N = 43). We assessed prevalence of atopy, asthma, FA, CRS/ROM, and PID. Innate immune responses were assessed by measuring production of proinflammatory and counter-regulatory cytokines by peripheral blood mononuclear cells (PBMCs) in response to agonists of Toll-like receptors (TLRs), with or without pre-treatment of lipopolysaccharide (LPS), a TLR4 agonist.

**Results:**

Non-IgE mediated FA was equally prevalent in both ASD test and ASD control groups, occurring at higher frequency than in the non-ASD controls. Allergic rhinitis, atopic/non-atopic asthma, and atopic dermatitis were equally prevalent among the study groups except for the CRS/ROM group in which non-atopic asthma was more prevalent (52.6%). CRS/ROM and specific polysaccharide antibody deficiency (SPAD) were more prevalent in the ASD test group than in the ASD control, FA, and normal control groups: 23.1% vs. < 5% for CRS/ROS and 19.2% vs. < 1% for SPAD. However, CRS/ROM patients had the highest prevalence of SPAD (34.2%). When compared to ASD and normal case controls, PBMCs from 19 non-SPAD, ASD test group children produced: 1) less IL-1β with a TLR7/8 agonist, less IL-10 with a TLR2/6 agonist, and more IL-23 with a TLR4 agonist without LPS pre-treatment, and 2) less IL-1β with TLR4/7/8 agonists with LPS pre-treatment. These are cytokines associated with the neuro-immune network.

**Conclusion:**

Clinical features of the ASD test group were not associated with atopy, asthma, FA, or PID in our study but may be associated with altered TLR responses mediating neuro-immune interactions.

## Background

Autism spectrum disorder (ASD) is a complex developmental disorder encompassing a heterogeneous patient population. It is generally agreed that there are at least two types of ASD with regard to disease development; abnormal cognitive development evident from birth (classical autism) and developmental regression, usually between 18–24 months of age, following apparent normal development (regressive autism) [1].

In addition to behavioral symptoms, co-morbid clinical conditions such as gastrointestinal (GI) symptoms are frequently noted in ASD children. A high prevalence of GI symptoms, which often improve after dietary intervention, has been reported by parents. This has led to speculation that there may be a high prevalence of food allergy (FA) in ASD children. Although IgE-mediated FA does not appear to be prevalent in ASD children, our previous study indicated a higher prevalence of non-IgE mediated FA (NFA) in ASD children. Specifically, our results revealed increased tumour necrosis factor-α (TNF-α) production against cow's milk protein along with correlating clinical features consistent with NFA [2]. In these ASD children with NFA, we also observed excessive production of TNF-α in response to LPS, an agonist of Toll-like receptor 4 (TLR4) [3].

Apart from FA, we have also encountered a number of ASD children who suffer from recurrent infection (typically viral syndromes) accompanied by exacerbations of behavioral symptoms (hyperactivity, temper tantrums, irritability and self-stimulatory behaviors) despite good responses to dietary intervention. Such behavioral changes were pointed out by teachers/therapists/care takers independent of parents. Immune insult via microbial infection caused by various pathogens appears to counter-act beneficial effects of behavioral, dietary, and other intervention measures in these ASD children. However, atopy or primary immunodeficiency (PID) does not appear to be a major factor in these children. The above-described clinical observations led us to hypothesize that in these ASD children, antigen non-specific (innate) immune responses are altered, leading to dysregulated neuro-immune interactions apart from PID or atopy.

Among 133 ASD children evaluated in our clinic, we identified 26 ASD children with the above-described clinical features (ASD test group). Interestingly, they are all documented to have shown regression at the onset of symptoms. In this study, we assessed prevalence of atopy, FA, CRS/ROM, and PID in the ASD test and control groups as well as in the non-ASD control groups (FA, CRS/ROM, and normal controls). In addition, we assessed responses to a panel of TLR agonists in 19 ASD test group children and compared these to responses for ASD and normal case controls.

## Methods

### Study Subjects

Study subjects included children with ASD, CRS/ROM, and FA that were referred to the Pediatric Allergy/Immunology Clinic at our institution from April, 2005 through May, 2008. These children were evaluated for atopic disorders, FA, and PID as medically indicated. They were also enrolled in institutional review board- (IRB-) approved study protocols that involved additional immunological evaluation of innate/adaptive immune responses. Control children were recruited from the same clinic and also from the General Pediatrics Clinic, and were evaluated for innate immune responses and atopy through their participation in established study protocols. Control children were children with no major medical issues affecting major organ systems, no documented behavioural problems, and age-appropriate cognitive activity as determined by his/her primary physician's evaluations and reported school performances obtained from chart review and from histories taken from their parents.

Demographic data of the study subjects are summarized in Table 1. Consistent with previous reports, the male/female ratio is significantly high in ASD children [4]. Normal control children also included higher numbers of males, reflecting our efforts to recruit normal control subjects matching the test group ASD children in age and sex. None of our study subjects were diagnosed with celiac disease or inflammatory bowel diseases.

**Table 1:** Demographics of the study subjects

Study Group	Number	Age (yr) Median (range)	Sex (male: female)	Race
ASD^1^				
Test	26	7.6 (2.3–13.4)	25:1	W^2 ^24, H 1, AA 0, A 1
Control	107	4.8 (1.5–17.3)	92:15	W 77, H 7, AA 11, A 9, other 3

CRS/ROM	38	6.8 (1.0–17.8)	27:11	W 12, H 16, AA 8, A 2

FA	24	2.5 (1.0–13.7)	18:6	W 12, H 4, AA 4, A 4

Control	43	7.0 (1.0–13.8)	31:12	W 31, H 6, AA 3, A 3

^1 ^ASD test group patients were diagnosed with autism (N = 21), pervasive developmental disorder not otherwise specified (PDD-NOS) (N = 4), and ASD (not categorized as autism or PDD-NOS due to age) (N = 1). ASD control group patients were diagnosed with autism (N = 70), PDD-NOS (N = 27), and ASD (not categorized as autism or PDD-NOS due to age) (N = 10).

^2 ^Abbreviations: W Caucasian, H Hispanic, AA African American, A Asian.

#### ASD diagnosis

ASD diagnosis was made or ascertained by DSM-IV (Diagnostic and Statistical Manual of Mental Disorders IV) criteria, ADI-R (Autism Diagnostic Interview-Revised), and/or ADOS (Autism Diagnostic Observational Schedules). ASD diagnosis was made by board-certified developmental pediatricians and/or pediatric neurologists/psychiatrists. The ADI-R and ADOS were administered by certified examiners from various autism centers including the ones at our institution.

The ASD test group was defined by having 1) frequent infections (more than 6/year documented by a physician) with poor responses to treatment measures such as the first line antibiotics and 2) at least 3 occurrences of changes in behavioral symptoms and/or loss of cognitive skills following infection, documented independently by caretakers, teachers, and therapists. Parents of the ASD test group reported repeated set backs of their children's progress at home and at school due to loss of acquired skills and interference from worsening behavioral issues. It is of note that all the ASD test group subjects were documented to have normal growth and development that was followed by major regression between 15–30 months of age. In most cases, this regression followed some type of immune insult (typically a viral syndrome.)

#### Diagnosis of atopic disorders

Allergic rhinitis (AR), allergic conjunctivitis (AC), and atopic dermatitis (AD) were diagnosed with positive skin-prick test reactivity and/or presence of allergen-specific IgE accompanied by clinical features consistent with AR, AC, or AD [5-7]. Asthma diagnosis was based on NIH guideline criteria [8]. Asthma without skin test reactivity and/or allergen-specific IgE antibody was categorized as non-atopic asthma[5]

#### Diagnosis of NFA

NFA to common dietary proteins (cow's milk protein, wheat, and soy) was diagnosed using the following criteria: 1) presence of objective GI symptoms (diarrhea, loose stool, and constipation) that resolved with avoidance of causative DPs, 2) delayed (more than 6 h) recurrence of GI symptoms following exposure to offending food after resolution of GI symptoms, and 3) cellular immune reactivity to offending dietary proteins defined as production of TNF-α and/or IL-12 greater than 1 standard deviation above control mean value by PBMCs after stimulation with causative dietary proteins [2].

#### Evaluation of ROM and CRS

ROM was defined as physician-diagnosed OM occurring more than 6 times/year. Most of the ROM patients referred to our clinic were unresponsive to surgical interventions (installment of pressure-equalizing tube and tonsillectomy/adenoidectomy). CRS was diagnosed with documentation of sinus inflammation by imaging studies (computerized tomography) despite prior prolonged/repeated courses of antibiotic therapy (more than 12 weeks) [9].

#### Evaluation of primary immunodeficiency

Presence of PID was evaluated in those patients with clinical features suggesting immune abnormalities such as recurrent or prolonged infections unresponsive to conventional treatment. Our routine immunological workup includes the following: serum levels of immunoglobulin (Ig), IgG subclass, and antibody titers to recall antigens; enumeration of peripheral blood T and B cell subsets, and T cell responses to mitogens and recall antigens.

For evaluation of innate immune responses, blood samples were collected following obtainment of the IRB-approved signed consent forms. At the time of venipuncture, all the study subjects were afebrile, were not on antibiotics, and had no evidence of acute microbial illnesses by physical examination.

### Cell cultures

PBMCs were isolated by Ficoll-Hypaque density gradient centrifugation. Innate immune responses were assessed by incubating PBMCs (10^6 ^cells/ml) overnight with TLR4 agonist (LPS; 0.1 μg/ml, GIBCO-BRL, Gaithersburg, MD), TLR2/6 agonist (zymosan; 50 μg/ml, Sigma-Aldrich, St. Luis, Mo), and TLR7/8 agonist (CL097, water-soluble derivative of imidazoquinoline, 20 μM, InvivoGen, San Diego, CA) in RPMI 1640 with additives as described before [10]. We then measured the levels of proinflammatory (TNF-α, IL-1β, IL-6, IL-12p40, IL-23) and counter-regulatory (IL-10, sTNFRII, and TGF-β) cytokines in the culture supernatant. When testing the effects of LPS pre-treatment, PBMCs were incubated overnight with LPS (0.1 μg/ml), the medium replaced, and then re-stimulated with the above-described TLR agonists.

All cytokine levels were measured by an enzyme-linked immunosorbent assay, using OptEIA™ Reagent Sets (BD Pharmingen, San Diego, CA) for TNF-α, IL-1β, IL-6, IL-10, IL-12p40 and ELISA reagent set (R & D, Minneapolis, MN) for sTNFRII and TGF-β. IL-23 ELISA kit was purchased from eBioscience (San Diego, CA). Intra- and inter-variations of cytokine levels were less than 5%.

### Statistics

For comparison of test values with control values, a Wilcoxon signed rank test was used. For comparison of values of multiple groups, a Kruskall-Walls test was used. For case control studies, a paired Wilcoxon signed rank test was used. Assessment of difference of frequency was tested with a Chi square (χ^2^) test. These tests were performed using R.2.5.1 (R-Development Core Team 2007). A p value of < 0.05 was considered to be statistically significant.

## Results

### Prevalence of atopy, FA, CRS/ROM, and PID in the study subjects

Table 2 summarizes the prevalence of atopy, FA, CRS/ROM, and PID in all of the study groups.

**Table 2:** Prevalence of atopy, food allergy, CRS/ROM and primary immunodeficiency in study subjects

Group	AR+AC	Asthma		AD	FA		CRS & ROM	PID^2^
		Atopy+	Atopy -		IgE	non-IgE		
ASD^1^								
Test^3^	7/26 (26.9%)	2/26 (7.7%)	2/26 (7.7%)	1/26 (3.8%)	2/26 (7.7%)	23/26 (88.5%)	6/26^5^ (23.1%)	5/26 (19.2%)
Control	23/107 (21.5%)	4/107 (2.4%)	11/107 (10.3%)	6/107 (5.6%)	6/107 (5.6%)^4^	82/107 (76.6%)	5/107 (4.7%)	1/107 (0.9%)

CRS & ROM	9/38 (23.7%)	9/38^6^ (23.7%)	20/38^6^ (52.6%)	0	0	7/38 (18.4%)	38/38 (100%)	13/38 (34.2%)

FA	5/24 (20.8%)	2/24 (8.3%)	2/24 (8.3%)	2/24 (8.3%)	3/24 (12.5%)	22/24 (91.7%)	1/24 (4.3%)	1/24^2^ (4.3%)

Control^7^	8/43 (18.6%)	6/43 (14.0%)	2/43 (4.7%)	2/43 (4.7%)	0	0	0	0

^1 ^Abbreviations: AC allergic conjunctivitis, AD atopic dermatitis, AR allergic rhinitis, ASD autism spectrum disorders, CRS chronic rhinosinusitis, FA food allergy, PID primary immunodeficiency, ROM recurrent otitis media.

^2 ^PID; ASD test/control groups: all diagnosed as specific Ab deficiency against polysaccharide Ags (SPAD), CRS/ROM group: 9 SPAD, 2 XLA, 1 dysmotile cilia syndrome, FA group: 1 patient was diagnosed as IgA deficiency. All the ASD test group children diagnosed with SPAD suffered from CRS/ROM.

^3 ^Seizure disorders were reported in 4/26 (15.4%) ASD-Test and 2/107 (1.9%) ASD-control groups, respectively.

^4 ^IgE mediated food allergy in ASD control group; all allergy to peanuts/tree nuts.

^5 ^Frequency of CRS/ROM and PID were higher in the ASD test group (p = 0.0334 and p = 5.762e-07, respectively, by χ^2 ^test).

^6 ^Frequency of atopic asthma and non-atopic asthma is higher in CRS/ROM children than other study groups (p = 0.00722 and p = 8.885e-10, respectively, by χ^2 ^test).

^7 ^Control children diagnosed with asthma had either mild intermittent or mild to moderate persistent asthma and seldom required oral steroid burst.

Despite the fact that many parents of ASD children reported that their children suffered from 'allergies', the prevalence of atopic disorders (AR+AC, AD, and atopic asthma) in the ASD test and ASD control groups were similar to that of the general population and to our normal controls [11]. CRS is a major trigger for asthma [12] and, not surprisingly, CRS/ROM patients were distinguished by a high frequency of atopic and non-atopic asthma as compared to other study groups (Table 2). In FA children, prevalence of atopy was not high but this may reflect their young age. AR+AC, AD, and asthma frequency in control children were equivalent to that in the general population [11]. These results indicate that atopy is not closely associated with clinical features of the ASD test group.

Consistent with the high prevalence of GI symptoms in ASD children, non-IgE-mediated, but not IgE-mediated, FA was observed at a high frequency in both ASD test and ASD control groups (Table 2). In contrast, despite frequent antibiosis, which causes GI symptoms by disrupting commensal flora and may subsequently predispose the subjects to FA, NFA was not as prevalent in CRS/ROM children as in the ASD children (p = 4.048e-11 by χ^2 ^test) (Table 2). These results indicate that FA is not closely associated with the clinical characteristics of the ASD test group.

The ASD test group children showed higher frequency of CRS/ROM and PID than did the ASD control group (Table 2). However, CRS/ROM patients showed the highest frequency of PID [mainly specific polysaccharide antibody deficiency (SPAD)] (Table 2). More importantly, diagnosis of these diseases alone does not explain the frequent infections and subsequent changes in behavioral symptoms in the majority of ASD test group children (Table 2).

### Evaluation of innate immune responses in the test group ASD children

We evaluated innate immune responses by assessing responses to TLR agonists that are important for airway and gut mucosal immunity in the ASD test group, excluding subjects diagnosed with SPAD. Results were compared with those obtained in ASD and normal case controls. Demographic summaries of ASD test-group subjects and of ASD and normal case controls are shown in Table 3. Since the ASD test group patients were all documented to have an initial regression, we selected ASD case controls who also had documented initial regression. We were not able to obtain such case controls in the FA and CRS/ROM study groups mainly due to age-limitations.

**Table 3:** Summary of matched cases

Study group	Age (yr) Median (range)	Atopy	Asthma
ASD-test^1^	6.4 (3.3–11.8)	5	3

ASD-control^2^	6.7 (3.2–12.1)	4	3

Normal control	6.4 (3.3–12.5)	5	4

^1 ^In ASD-test group, they are diagnosed with autism (N = 15) and PDD-NOS (N = 4) and the ASD case controls were matched with ASD diagnosis in addition to age and sex.

^2 ^All the ASD case controls were documented to have developmental regression at the onset of ASD symptoms as were the ASD test group children.

**Figure 1:**
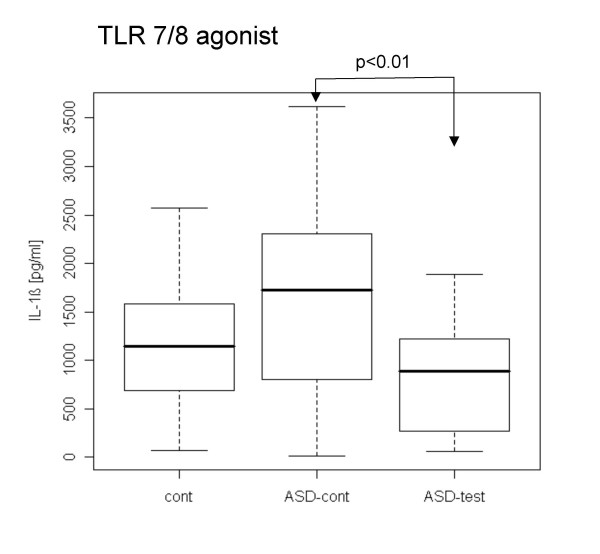
**Lower IL-1β production with a TLR7/8 agonist in the ASD test group without LPS pre-treatment**. In all experiments shown in Figures 1, 2, 3, 4, 5 and 6, PBMCs were incubated overnight with TLR2/6, TLR4 or TLR7/8 agonists, and cytokine production was examined by ELISA. The results were expressed using a boxplot: The "I" bar marks the range of TGF-β and IL-10 levels and the thick black line marks the median. The box illustrates where the 'interquartile range' falls. 'Outliers' are marked separately as 'o'. IL-1β production with a TLR7/8 agonist was lower in the ASD test group than in the ASD case controls.

**Figure 2:**
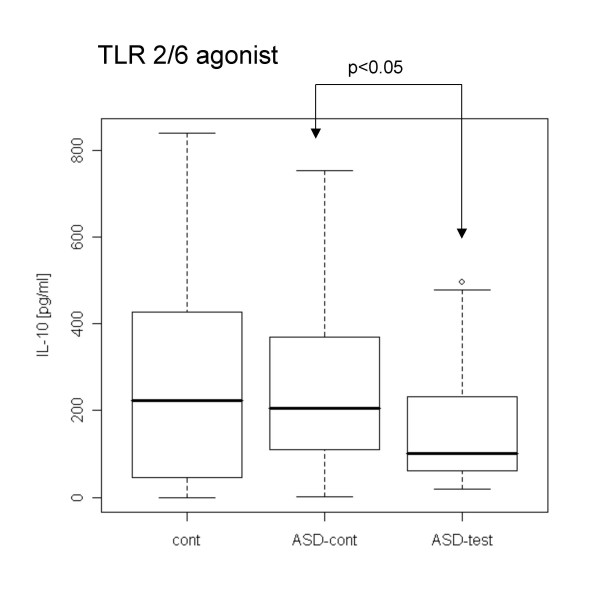
**Lower IL-10 production with a TLR2/6 agonist in the ASD-test group without LPS pre-treatment**. IL-10 production with a TLR2/6 agonist was lower in the ASD-test group than in the ASD case controls.

**Figure 3:**
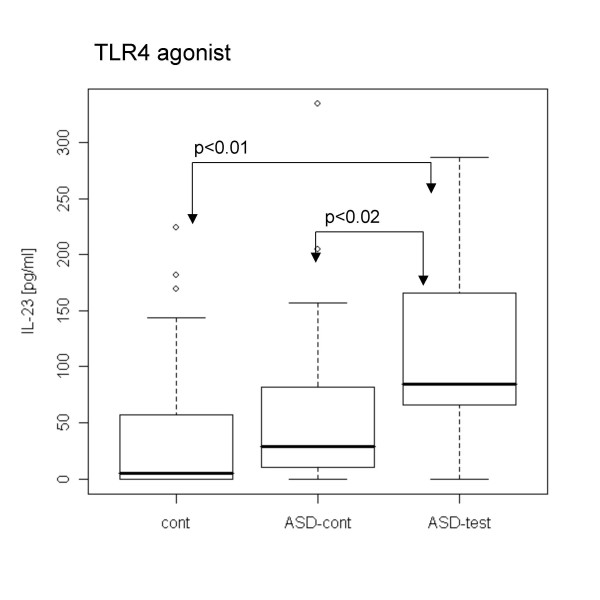
**Higher IL-23 production with a TLR4 agonist in the ASD test group without LPS pre-treatment**. IL-23 production with a TLR4 agonist was lower in the ASD test group than in the ASD and normal case controls.

When PBMCs were stimulated with TLR agonists without LPS pre-treatment, PBMCs from the ASD test group revealed lower IL-1β production with a TLR7/8 agonist and lower IL-10 production with a TLR2/6 agonist than did ASD control cells (Figs. 1, 2). In contrast, the ASD test group revealed higher IL-23 production with the TLR4 agonist than control groups (Fig. 3).

**Figure 4:**
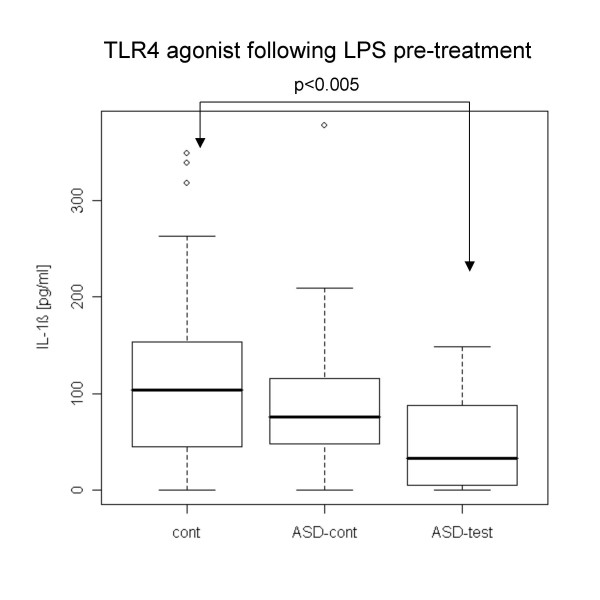
**Lower IL-1β production with a TLR4 agonist in the ASD test group following LPS pre-treatment**. In experiments shown in Figures 4, 5 and 6, PBMCs were treated with LPS (0.1 μg/ml) overnight and re-stimulated with TLR agonists as described in Figure 1 after replacing the culture medium. IL-1β production was lower in the ASD test group in this setting than in the normal case controls.

**Figure 5:**
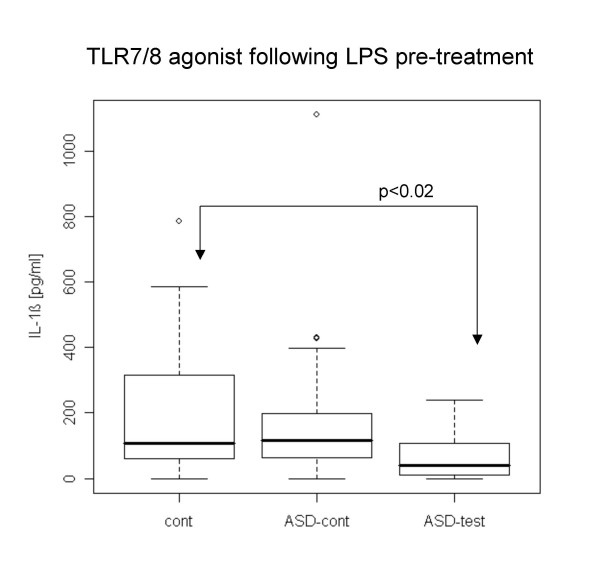
**Lower IL-1β production with a TLR7/8 agonist in the ASD test group following LPS pre-treatment**. IL-1β production was lower in the ASD test group following LPS pre-treatment in the ASD test group than in the normal case controls.

**Figure 6:**
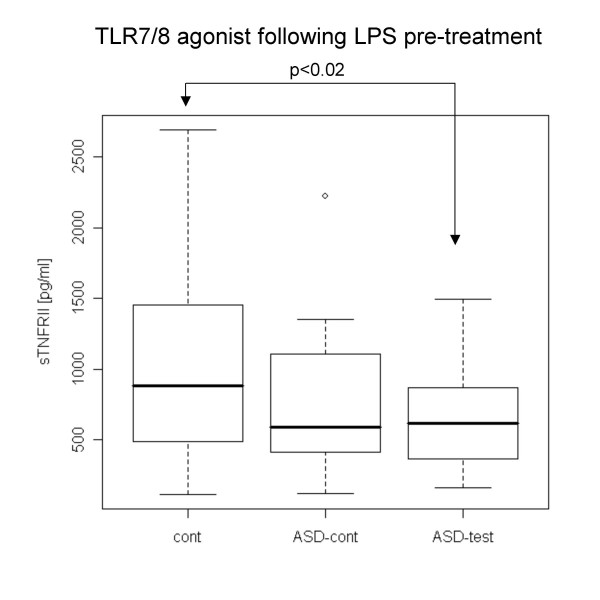
**Lower sTNFRII production with a TLR7/8 agonist in the ASD test group following LPS pre-treatment**. sTNFRII production with a TLR7/8 agonist was lower in the ASD test group than in normal case controls.

Following LPS pre-treatment, the ASD test group revealed lower IL-1β production with TLR4 and TLR7/8 agonists (Fig. 4, 5). The ASD control group did not reveal such differences. sTFNRII production with a TLR7/8 agonist following LPS pre-treatment was lower in the ASD test group than in normal controls, and ASD controls also revealed this tendency except for 2 outliers (Fig. 6)

## Discussion

Subtle immune abnormalities in ASD children have been reported by many researchers [13-15]. However, results are rather variable partly due to the small number of study subjects, heterogeneous patient populations, and lack of careful immunological characterization of the study subjects in these studies. Moreover, reported results are conflicting; some studies indicate type 1 T-helper (Th1)-skewed responses while other studies indicate Th2-skewed responses [13-16]. A high prevalence of GI symptoms in autistic children also raised possibilities of GI autoimmune conditions and FA. However, to date, the role of the immune system in the onset and progression of ASD remains unclear.

The effects of environmental factors in genetically vulnerable individuals have also been implicated in the development of autism. There is ample evidence indicating that there is increased oxidative stress in autistic children [17]. The 'redux/methylation hypothesis' postulates that environmental factors and genetic factors play pathogenic roles in some autistic children [17]. It has been postulated that exposure to xenobiotics in subjects with a genetic predisposition for impaired methylation and/or increased susceptibility to oxidative stress may result in the neurological deficits observed in certain ASD children [18,19]. Oxidative stress also activates innate immunity. Interestingly, the presence of low-grade chronic inflammation has been reported in brain tissue of individuals with autism [20,21]. This 'redux/methylation hypothesis' may also be associated with skewed Th1 or Th2 responses in certain conditions [22]. However, how such genetic/environmental factors modulate the immune system in autistic children is not well understood.

For a number of ASD children, parents report frequent infections [recurrent pharyngitis and viral syndromes, ROM, and CRS] and an unusually prolonged course of such illnesses. Following microbial infection, parents repeatedly describe exacerbations of behavioral symptoms and even loss of previously acquired cognitive skills, an occurrence also documented by care-takers/teachers/therapists. This study focused on this subset of ASD children (ASD test group) as defined in the Methods section. Our first hypothesis is that clinical features of the ASD test group are not associated with atopy, asthma, FA, or PID.

To test this hypothesis, we retrospectively reviewed 133 ASD children referred to the Pediatric Allergy/Immunology Clinic for evaluation of atopy/immune abnormalities. Given the nature of our clinic, the ASD children reviewed may have had more medical issues than ASD children in general, as evidenced by their need to visit the clinic. Nevertheless, it is advantageous to analyze these children, since extensive data had already been generated by thorough allergy and immune workups. We also have the advantage of having control non-ASD children with chronic airway or GI inflammation who underwent a similar workup (control CRS/ROM and FA groups) that enabled us to address the presence of any distinguishing clinical features in the ASD test group.

Prevalence of atopic disorders and asthma was equivalent to that reported in general population in both test and control ASD children. A lower prevalence of atopy in the control FA patients may reflect their young age, since atopic disorders develop with age. Likewise, a younger median age of the control ASD children indicates that atopic diseases may develop with age. Nevertheless, given the frequency of atopic disorders in the ASD test group, who are slightly older in median age than the ASD controls, it is very unlikely that atopy is associated with the clinical features of the ASD test group.

NFA was highly prevalent in both ASD groups, consistent with the high prevalence of GI symptoms in ASD children (Table 2). As summarized in Table 2, a major difference in the clinical features of the test vs. control ASD children was the higher frequency of CRS/ROM and SPAD in the ASD test group children. However, diagnoses of CRS/ROM and SPAD were only seen in 6/26 and 5/26 of the ASD test group children, respectively. All of the ASD test group children with SPAD suffered from CRS/ROM (Table 2). SPAD was most prevalent in CRS/ROM children (Table 2). These findings taken together suggest that the distinguishing clinical features of the ASD test group are unlikely to be associated with FA, asthma, or SPAD in most of the ASD test group children.

In CRS patients, it was postulated that transmission of inflammatory mediators via post-nasal dripping may be associated with chronic GI irritation and predispose them to FA [23]. It was also speculated that frequent antibiosis and resultant dysbiosis can disrupt gut mucosal immune homeostasis, resulting in sensitization to food proteins. However, our results revealed a higher prevalence of NFA in ASD children than in CRS/ROM children, who were likely to have had more frequent antibiosis than ASD children (Tables 2). Asthma was prevalent in CRS/ROM children, which is not surprising since CRS is one of the major triggers of asthma [12]. Our findings indicate CRS/ROM children do not display clinical features similar to those observed in ASD test or ASD control children, making it unlikely that CRS/ROM account for the high prevalence of NFA in ASD children in our study.

It is of note that we did not find a high prevalence of known PID in control ASD children, although the test group ASD children revealed a few cases of SPAD (Table 2). Therefore, extensive immune workup focusing on PID is likely to yield negative results in most ASD children, especially those without concerning clinical features, as reported previously [24].

In summary, our results support our initial hypothesis that the distinct clinical features of our ASD test group are not associated with atopy, asthma, FA, or presence of known PID. However, our results have limitations. The number of the subjects and a potentially skewed population due to the fact that the ASD study subjects were those evaluated in the Pediatric Allergy/Immunology Clinic have some bearing on our results. A larger population study will be informative to further elaborate our initial findings.

In this study, we identified 19 ASD test group children without SPAD among 133 ASD children (14.3%). Pathogens affecting these ASD test group children appeared various, indicating the possibility of impaired antigen-independent immune responses – innate immunity. Innate immunity mounts the first line, antigen-independent immune defense by recognizing microbial by-products or those from damaged tissue cells via pattern recognition receptors including TLRs [25-27]. TLR-mediated responses lead to the production of various soluble mediators that can signal the brain [28-31]. Such signalling events help the central nervous system restore autonomic homeostasis and provide inhibitory regulatory signals to prevent excessive immune responses [28]. Subtle changes in innate immune responses can affect the intricate neuro-immune network that is mediated by innate immunity. Since NFA prevalence was similar in both the ASD groups, it was the frequent occurrence of viral syndromes in the ASD test group children that led us to our 2^nd ^hypothesis. Namely, we theorized that TLR responses, especially those sensing viral by-products, are altered in the ASD test group.

Among TLRs, TLR2 is important for sensing encapsulated bacteria and intracellular pathogens such as mycobacterium [32,33]. TLR4 is important for sensing gram negative bacteria, common found in the GI tract [32]. TLR7/8 are important for sensing single stranded RNA derived from RNA viruses, which are common causes of childhood viral infections including measles, rhinovirus (the most common cause of 'cold'), and influenza [34]. This study assessed responses to TLR2/6, 4, and 7/8 agonists by measuring production of a panel of cytokines in the test group ASD children without SPAD and comparing those to children with ASD and to normal case controls. It is important to mention that since many FA patients outgrow this condition with age, the FA control children are younger than the ASD children evaluated in the study. In addition, the number of non-ASD controls with FA or CRS/ROM is low due to the fact that the study was limited to patients evaluated/treated in our clinic. Thus the study is lacking non-ASD case controls with FA or CRS/ROM for evaluation of innate immune responses.

Several abnormalities were found in the ASD test group: lower production of IL-6 with the TLR2/6 agonist, lower production of IL-1β with the TLR7/8 agonist, and higher production of IL-23 with the TLR4 agonists than case controls in the absence of LPS pre-treatment. These cytokines which were found to be altered in production in the ASD test group are the key regulators in the neuro-immune network [28,35,36]. Thus our findings indicate that children in the ASD test group may be less capable of controlling microbial infection in the initial stages, leading to ineffective signalling to the brain. It is also intriguing to find increased production of IL-23 with the TLR4 agonist in the ASD test, since IL-23 is associated with development and maintenance of Th17 cells, a recently defined T-helper cell subset [37,38]. It is of note that Th17 cells are implicated in various autoimmune and chronic inflammatory diseases including multiple sclerosis and inflammatory bowel diseases [37-39]. Thus the ASD test group children may be more prone to Th17-mediated inflammatory responses.

When immune cells were pre-treated with LPS, a TLR4 agonist, subsequent TLR responses were suppressed; this phenomenon is called LPS tolerance [40]. This is thought to be important for immune homeostasis in the gut, and especially in the colon where bacteria exist at high density and hence innate immune cells in the mucosa are likely to have frequent exposures to endotoxins (LPS) [40]. We found lower IL-1β production by PBMCs with TLR4 or TLR7/8 agonists following LPS pre-treatment in the ASD test group. IL-1β is one of the major mediators of the neuro-immune network for maintenance of homeostasis [35,36,41]. Thus suboptimal production of IL-1β may impair neuro-immune signaling, while excessive IL-1β can be toxic to the brain [35,41,42]. Our finding may indicate a risk of suboptimal neuro-immune signalling in the ASD test group.

sTNFRII is an important counter-regulatory cytokine for TNF-α as made evident by the marked therapeutic effects of exogenous sTNFRII in the treatment of certain autoimmune diseases [43]. We also found lower sTNFRII production by PBMCs with TLR 7/8 agonist following LPS pre-treatment in the ASD test group, indicating a possibility of blunted counter-regulatory measures in the ASD test group at the time of viral infection. However, sTNFRII production in this condition also appeared to be lower in the ASD control group except for 2 high outliers. Thus this finding may not be specific for the ASD test group children.

Taken together, our findings of altered TLR7/8 responses with or without endotoxin (LPS) pre-treatment may indicate a predisposition to recurrent viral infections in the ASD test group as compared to ASD and non-ASD case controls. The above-described, altered TLR responses may also affect neuro-immune interactions in the test group. Our findings may be useful further defining this subset of ASD children in larger scale studies.

## Conclusion

Our results indicate the presence of a distinct subset of ASD children. This subset, the ASD-test group, is characterized by frequent infections and by recurrent loss of previously acquired cognitive skills with worsening behavioral symptoms following infection. Clinical features of this subset were not associated with atopy, asthma, FA, or PID in this study but may be associated with altered TLR responses important for neuro-immune interactions.

## Abbreviations

AC: allergic conjunctivitis; AD: atopic dermatitis; AR: allergic rhinitis; ASD: autism spectrum disorder; CRS: chronic rhinosinusitis; FA: food allergy; GI: gastrointestinal; Ig: immunoglobulin; IL: interleukin; LPS: lipopolysaccharide; NFA: non-IgE mediated food allergy; PBMCs: peripheral blood mononuclear cells; PID: primary immunodeficiency; ROM: recurrent otitis media; SPAD: specific polysaccharide antibody deficiency; sTNFRII: soluble TNF receptor II; TGF: transforming growth factor; Th: T-helper; TLR: toll like receptor; TNF: tumor necrosis factor.

## Competing interests

The authors declare that they have no competing interests.

## Authors' contributions

HJ designed this project, obtained approval of IRB, recruited study subjects and obtained blood samples. HJ also reviewed the clinical data, analyzed the data of TLR responses, and prepared most of the manuscript.

LG carried out all the assays of TLR responses and helped HJ to analyze data of TLR responses.

AC helped HJ for obtainment of the IRB approval and recruitment of the study subjects and sample obtainment as a clinical coordinator in the Pediatric Allergy/Immunology Clinic. HQ participated in recruitment of CRS/ROM patients and also helped HJ in retrospective review of the study subjects with regard to CRS/ROM as a pediatric otolaryngologist.

## References

[B1] Lainhart JE, Ozonoff S, Coon H, Krasny L, Dinh E, Nice J, McMahon W (2002). Autism, regression, and the broader autism phenotype. Am J Med Genet.

[B2] Jyonouchi H, Geng L, Ruby A, Reddy C, Zimmerman-Bier B (2005). Evaluation of an association between gastrointestinal symptoms and cytokine production against common dietary proteins in children with autism spectrum disorders. J Pediatr.

[B3] Jyonouchi H, Geng L, Ruby A, Zimmerman-Bier B (2005). Dysregulated innate immune responses in young children with autism spectrum disorders: their relationship to gastrointestinal symptoms and dietary intervention. Neuropsychobiology.

[B4] Fombonne E (2005). Epidemiology of autistic disorder and other pervasive developmental disorders. J Clin Psychiatry.

[B5] Nassef M, Shapiro G, Casale TB (2006). Identifying and managing rhinitis and its subtypes: allergic and nonallergic components–a consensus report and materials from the Respiratory and Allergic Disease Foundation. Curr Med Res Opin.

[B6] Butrus S, Portela R (2005). Ocular allergy: diagnosis and treatment. Ophthalmol Clin North Am.

[B7] Akdis CA, Akdis M, Bieber T, Bindslev-Jensen C, Boguniewicz M, Eigenmann P, Hamid Q, Kapp A, Leung DY, Lipozencic J (2006). Diagnosis and treatment of atopic dermatitis in children and adults: European Academy of Allergology and Clinical Immunology/American Academy of Allergy, Asthma and Immunology/PRACTALL Consensus Report. J Allergy Clin Immunol.

[B8] L National Heart, and Blood Institute; National Asthma Education and Prevention Program (2002). Expert panel report 2 update:guidelines for the diagnosis and management of asthma. L National Heart, and Blood Institute ed.

[B9] Steele RW (2006). Rhinosinusitis in children. Curr Allergy Asthma Rep.

[B10] Jyonouchi H, Sun S, Itokazu N (2002). Innate immunity associated with inflammatory responses and cytokine production against common dietary proteins in patients with autism spectrum disorder. Neuropsychobiology.

[B11] Ronchetti R, Rennerova Z, Barreto M, Villa MP (2007). The prevalence of atopy in asthmatic children correlates strictly with the prevalence of atopy among nonasthmatic children. Int Arch Allergy Immunol.

[B12] Corren J, Kachru R (2007). Relationship between nonallergic upper airway disease and asthma. Clin Allergy Immunol.

[B13] Ashwood P, Water J Van de (2004). Is autism an autoimmune disease?. Autoimmun Rev.

[B14] Ashwood P, Water J Van de (2004). A review of autism and the immune response. Clin Dev Immunol.

[B15] Cohly HH, Panja A (2005). Immunological findings in autism. Int Rev Neurobiol.

[B16] Ashwood P, Wills S, Water J Van de (2006). The immune response in autism: a new frontier for autism research. J Leukoc Biol.

[B17] Deth R, Muratore C, Benzecry J, Power-Charnitsky VA, Waly M (2008). How environmental and genetic factors combine to cause autism: A redox/methylation hypothesis. Neurotoxicology.

[B18] Herbert MR, Russo JP, Yang S, Roohi J, Blaxill M, Kahler SG, Cremer L, Hatchwell E (2006). Autism and environmental genomics. Neurotoxicology.

[B19] James SJ, Melnyk S, Jernigan S, Cleves MA, Halsted CH, Wong DH, Cutler P, Bock K, Boris M, Bradstreet JJ (2006). Metabolic endophenotype and related genotypes are associated with oxidative stress in children with autism. Am J Med Genet B Neuropsychiatr Genet.

[B20] Pardo CA, Vargas DL, Zimmerman AW (2005). Immunity, neuroglia and neuroinflammation in autism. Int Rev Psychiatry.

[B21] Vargas DL, Nascimbene C, Krishnan C, Zimmerman AW, Pardo CA (2005). Neuroglial activation and neuroinflammation in the brain of patients with autism. Ann Neurol.

[B22] Riedl MA, Nel AE (2008). Importance of oxidative stress in the pathogenesis and treatment of asthma. Curr Opin Allergy Clin Immunol.

[B23] Liu T, Wang BQ, Zheng PY, He SH, Yang PC (2006). Rhinosinusitis derived Staphylococcal enterotoxin B plays a possible role in pathogenesis of food allergy. BMC Gastroenterol.

[B24] Stern L, Francoeur MJ, Primeau MN, Sommerville W, Fombonne E, Mazer BD (2005). Immune function in autistic children. Ann Allergy Asthma Immunol.

[B25] Kabelitz D, Medzhitov R (2007). Innate immunity–cross-talk with adaptive immunity through pattern recognition receptors and cytokines. Curr Opin Immunol.

[B26] Beutler B, Hoebe K, Georgel P, Tabeta K, Du X (2005). Genetic analysis of innate immunity: identification and function of the TIR adapter proteins. Adv Exp Med Biol.

[B27] Pasare C, Medzhitov R (2005). Toll-like receptors: linking innate and adaptive immunity. Adv Exp Med Biol.

[B28] Wrona D (2006). Neural-immune interactions: an integrative view of the bidirectional relationship between the brain and immune systems. J Neuroimmunol.

[B29] Dantzer R (2004). Innate immunity at the forefront of psychoneuroimmunology. Brain Behav Immun.

[B30] Glezer I, Simard AR, Rivest S (2007). Neuroprotective role of the innate immune system by microglia. Neuroscience.

[B31] Haddad JJ (2008). On the mechanisms and putative pathways involving neuroimmune interactions. Biochem Biophys Res Commun.

[B32] Turvey SE, Hawn TR (2006). Towards subtlety: understanding the role of Toll-like receptor signaling in susceptibility to human infections. Clin Immunol.

[B33] Miyake K (2007). Innate immune sensing of pathogens and danger signals by cell surface Toll-like receptors. Semin Immunol.

[B34] Uematsu S, Akira S (2007). Toll-like receptors and Type I interferons. J Biol Chem.

[B35] Gosselin D, Rivest S (2007). Role of IL-1 and TNF in the brain: twenty years of progress on a Dr. Jekyll/Mr. Hyde duality of the innate immune system. Brain Behav Immun.

[B36] Pace TW, Hu F, Miller AH (2007). Cytokine-effects on glucocorticoid receptor function: relevance to glucocorticoid resistance and the pathophysiology and treatment of major depression. Brain Behav Immun.

[B37] McGeachy MJ, Cua DJ (2008). Th17 cell differentiation: the long and winding road. Immunity.

[B38] Maloy KJ (2008). The Interleukin-23/Interleukin-17 axis in intestinal inflammation. J Intern Med.

[B39] Tzartos JS, Friese MA, Craner MJ, Palace J, Newcombe J, Esiri MM, Fugger L (2008). Interleukin-17 production in central nervous system-infiltrating T cells and glial cells is associated with active disease in multiple sclerosis. Am J Pathol.

[B40] Smith DW, Nagler-Anderson C (2005). Preventing intolerance: the induction of nonresponsiveness to dietary and microbial antigens in the intestinal mucosa. J Immunol.

[B41] Viviani B, Gardoni F, Marinovich M (2007). Cytokines and neuronal ion channels in health and disease. Int Rev Neurobiol.

[B42] Patel HC, Boutin H, Allan SM (2003). Interleukin-1 in the brain: mechanisms of action in acute neurodegeneration. Ann N Y Acad Sci.

[B43] Cobo-Ibanez T, Martin-Mola E (2007). Etanercept: long-term clinical experience in rheumatoid arthritis and other arthritis. Expert Opin Pharmacother.

